# Application of latent semantic analysis for open-ended responses in a large, epidemiologic study

**DOI:** 10.1186/1471-2288-11-136

**Published:** 2011-10-05

**Authors:** Travis D Leleu, Isabel G Jacobson, Cynthia A LeardMann, Besa Smith, Peter W Foltz, Paul J Amoroso, Marcia A Derr, Margaret AK Ryan, Tyler C Smith

**Affiliations:** 1 Deployment Health Research Department at Naval Health Research Center, 140 Sylvester Road, San Diego, CA, 92106, USA; 2 2MultiCare Research Institute, Tacoma, WA, 98415, USA; 3 Pearson Knowledge Technologies, 4940 Pearl East Circle, Suite 200, Boulder, CO, 80301, USA; 4 Naval Hospital Camp Pendleton, Box 555191, Camp Pendleton, CA, 92055, USA

## Abstract

**Background:**

The Millennium Cohort Study is a longitudinal cohort study designed in the late 1990s to evaluate how military service may affect long-term health. The purpose of this investigation was to examine characteristics of Millennium Cohort Study participants who responded to the open-ended question, and to identify and investigate the most commonly reported areas of concern.

**Methods:**

Participants who responded during the 2001-2003 and 2004-2006 questionnaire cycles were included in this study (*n *= 108,129). To perform these analyses, Latent Semantic Analysis (LSA) was applied to a broad open-ended question asking the participant if there were any additional health concerns. Multivariable logistic regression was performed to examine the adjusted odds of responding to the open-text field, and cluster analysis was executed to understand the major areas of concern for participants providing open-ended responses.

**Results:**

Participants who provided information in the open-ended text field (*n *= 27,916), had significantly lower self-reported general health compared with those who did not provide information in the open-ended text field. The bulk of responses concerned a finite number of topics, most notably illness/injury, exposure, and exercise.

**Conclusion:**

These findings suggest generalized topic areas, as well as identify subgroups who are more likely to provide additional information in their response that may add insight into future epidemiologic and military research.

## Background

Qualitative data can provide epidemiologists with invaluable information that cannot be captured by quantitative data alone. Open-ended survey responses are difficult to analyze quantitatively in a large-scale study due to time constraints and complexity of categorizing the responses in a consistent and unbiased way. Latent Semantic Analysis (LSA) provides a method for open-ended text analysis using sophisticated statistical and mathematical algorithms [1]. This method reveals subtle textual meaning using an automated approach that eliminates potential human bias and permits rapid coding of large amounts of data [2]. LSA is widely used in applications of information retrieval [1], spam filtering [3], and automated essay scoring [4]. To date, modest assessments of LSA's functionality for open-ended text responses have shown promising results [5], opening the field of large-scale application of this technique to areas such as epidemiologic survey research.

This investigation explores the use of LSA to analyze open-ended responses from Millennium Cohort Study participants collected from 2001-2006 to investigate important health concerns that may not be covered by the structured questionnaire. Participant responses may also add value to existing research by providing more insight into emerging areas of concern. Additionally, it may prompt suggestions for refining future versions of the questionnaire by including previously omitted topics. The use of LSA for efficient and standardized analysis of open-ended responses from large-scale studies such as the Millennium Cohort will further epidemiological research by allowing researchers to gain deeper insight of populations under study.

## Methods

### Population and data sources

This cross-sectional investigation is part of the larger Millennium Cohort Study, which was designed in the late 1990s to determine how military service may affect long-term health [6]. Those invited to participate in Panel 1 of the Millennium Cohort Study were randomly selected from all US military personnel, over sampling female service members, Reserve/National Guard service members, and those who had been previously deployed to southwest Asia, Bosnia, or Kosovo from 1998 through 2000, to ensure sufficient power to detect differences in smaller subgroups of the population. The probability-based sample, representing approximately 11.3 percent of the 2.2 million men and women in service as of October 2000, was provided by the Defense Manpower Data Center (DMDC) in California. Of the 77,047 individuals who enrolled (36 percent response rate) from July 2001 to June 2003 in Panel 1, 55,021 (71 percent follow-up rate) completed the first follow-up questionnaire between June 2004 and February 2006. In addition to Panel 1, the invited participants of Panel 2 were randomly selected from military personnel with 1 to 2 years of service as of October 2003, and 31,110 enrolled (25 percent response rate). Marines and women were over sampled in this panel in order to ensure sufficient power among women as well as the most likely group of combat deployers. This investigation began with 108,157 consenting participants who completed a questionnaire from either Panel 1 (baseline and/or follow-up) or Panel 2 baseline. Investigations of nonresponse to the first follow-up questionnaire found no appreciable bias as reflected by comparing measures of association for selected outcomes using complete case and inverse probability weighting [7]. Participants with missing covariate data were removed from analyses. Demographic and military-specific data were obtained from electronic personnel files maintained by DMDC. Variables included sex, birth date, highest education level, marital status, race/ethnicity, past deployment to southwest Asia, Bosnia, or Kosovo between 1998 and 2000, pay grade, service component (active duty and reserve/National Guard), service branch (Army, Navy, Coast Guard, Air Force, and Marine Corps), and occupations.

The questionnaire consisted of 67 questions, including the open-ended question that read, "Do you have any concerns about your health that are not covered in this survey that you would like to share". While other questions allowed for free form text input, they were designed to accommodate only brief responses. The open-ended question was designed for participants to include as much information as they wanted, over any subject they wished to discuss. The huge variance in response topics made simplistic dictionary analysis of the open-ended response untenable. In addition, dictionary based analyses are unable to account for polysemy, a situation where one word can have multiple meanings (e.g., *back *can mean *back pain, backwards, or previous in time*).

### Latent Semantic Analysis

LSA is a fully automatic mathematical/statistical technique for extracting and inferring meaningful relations from the contextual usage of words [8,9]. Using LSA software developed by Pearson Knowledge Technologies, lexical analysis was performed on the responses to the final question, which asks participants to share any other health concerns not covered in the structured instrument. This allowed for identifying semantic similarities among open text responses to determine clusters of responses with high contextual similarity (e.g., noting that "welding fumes" and "asbestos" have similar meaning within the context of this study). LSA overcomes the limitations of simple dictionary-based analysis because it determines meaning from contextual similarity, rather than human defined synonyms and related words.

The first step in applying LSA to the analysis of open-ended responses was to create a semantic space, "a mathematical representation of a large body of text[s]" [9], using a corpus of medical and military documents as well as the text of the questionnaire itself and the open-ended responses. The semantic space was generated from 1,862,972 medical and military documents comprising 435,456 unique terms. These documents included medical journal articles containing health related writings, military documents replete with jargon and geographical locations, plus common English language works. In addition, the open-ended responses were included in the semantic space in order to identify semantic similarities that would not exist outside the context of an open-ended response. To reduce complexity, the size of the semantic space was optimized by LSA to have n = 300 dimensions. Data were then filtered by removing responses that conveyed no information about the health of the participant (e.g., "No," "N/A," "I have nothing to say"). This removed entire responses from the analysis, an important distinction from the common tactic of employing a "stop list", which removes common words (e.g., "and", "the", etc.) from specific responses. In this analysis, every word in every response was considered for analysis; only the responses determined to convey no meaning were removed. Once identified, those individuals with meaningless responses (*n *= 33,951) were included in the group of participants who did not respond to the open-ended question. Upon human examination, 25 (0.1 percent) responses were originally classified as meaningless that were subsequently reclassified as meaningful. To investigate the number of responses misclassified as meaningful, a random sample of 250 responses originally classified as meaningful were reviewed by humans. Of these, only 5 (2.0 percent) were judged to be actual meaningless responses. Therefore, the classification method biased slightly toward categorizing responses as meaningful rather than the opposite. Implications of this small amount of misclassification are expected to have minimal effects on our study findings.

A set of 1025 clustering terms was created by selecting words from the meaningful responses that each appeared more than 70 times (excluding words in a high-frequency stop list; a stop list was not used in the creation of the semantic space). LSA was used to compute a dissimilarity measure by computing the cosine between each pair of terms in the set to produce a distance matrix. The set of terms was partitioned into 20 non-overlapping clusters using a variant of the k-means clustering algorithm, called the *pam *(for "partitioning around medoids") function from the R language *cluster *package. Twenty clusters were chosen since more than 20 clusters gave redundant or overlapping clusters, or clusters that were not relevant to the medical domain (e.g. measures of time, military terms). Fewer than 20 clusters did not provide sufficient separation into separate categories. Each cluster was represented by its medoid, the term most central in the cluster. Meaningful responses were assigned to clusters by computing the similarity between each response and each cluster medoid. If the cosine between a response and a medoid (representing the vector distance between a given response and the cluster medoid) was greater than 0.2, the response was assigned to that cluster. The clusters were then ranked based on how many responses they contained. The 20 clusters that accounted for the most responses were examined to determine their semantic meaning. However, not all of the top-20 clusters had discernable semantic meaning; some clusters appeared to be an artifact of the LSA technology (e.g., the cluster described by the following terms: a lot, don't, haven't, isn't, believed). For this exploratory analysis, the clusters without obvious semantic meaning were not included due to the difficulty determining the topic of concern. Responses could be assigned to multiple clusters, though this occurred infrequently. This analysis resulted in 24,181 (86.6 percent) of the 27,916 meaningful responses being assigned to at least one area of concern (represented by membership in a cluster).

### Statistical analysis

Descriptive and quantitative analyses of demographic characteristics among those who did and did not respond to the open-ended question were performed. Multivariable logistic regression modeling was used to investigate associations between demographic characteristics and whether they responded to the open-ended text question. A separate logistic regression model was run for Panel 1 baseline, Panel 1 follow-up, and Panel 2 baseline populations. All statistical data analyses were performed using SAS statistical software version 9.2 (SAS Institute Inc., Cary, NC).

## Results

The semantic space was generated from 1,862,972 medical and military documents comprising 435,456 unique terms using 300 dimensions. Of the 108,157 eligible participants, 19 were removed due to missing information for education and marital status, leaving 108,138 participants for analyses. Of the 108,138 participants in the study who completed 163,159 surveys from 2001-2006 (encompassing Panel 1 baseline and follow-up, and Panel 2 baseline), 61,507 surveys (37.7 percent) had a response in the open-ended field. There were 670 unique null patterns (indicating a meaningless response) identified, resulting in 33,591 of the open-ended responses (54.6 percent) being classified as having a meaningless response. Subsequently, 27,916 (45.4 percent of open-ended responses, 17.1 percent of all completed surveys) were classified with meaningful responses.

Table 1 describes characteristics of Millennium Cohort Study participants who responded to the open-ended question, stratified by panel and survey. Open-ended responders were generally representative of their overall panel characteristics. However, for all three groups, a higher proportion of open-ended responders were older, on active duty, Army members, and combat specialists. Education level did not have a significant effect on response to the open ended question. In addition, open-ended responders were more likely to self-report good, fair, or poor general heath compared with those who did not provide an open-ended response who were more likely to report very good or excellent health.

**Table 1:** Characteristics of Millennium Cohort Study Participants Who Provided a Meaningful Response for the Open-Ended Question

	**Panel 1 Baseline**		**Panel 1 Follow-up**		**Panel 2 Baseline**	
	
**Characteristic**	**All responders** ***n *= 77,042**	**Open-text responders^a^** ***n *= 14,692**	**All responders** ***n *= 55,021**	**Open-text responders^a^** ***n *= 8,937**	**All responders** ***n *= 31,096**	**Open-text responders^a^** ***n *= 4,287**
	
	**%^b^**	**%^b^**	**%^b^**	**%^b^**	**%^b^**	**%^b^**
	
Sex						
Male	73.2	73.2	73.3	72.8	61.6	63.4
Female	26.8	26.8	26.7	27.2	38.4	36.6
Birth year						
Before 1960	21.6	24.2	24.5	28.1	0.7	0.9
1960-1969	37.9	39.2	40.5	40.5	5.4	6.3
1970-1979	34.6	31.8	30.8	28.1	31.9	35.6
1980 or later	5.9	4.7	4.2	3.3	62.0	57.2
Education						
High school or less	48.9	48.9	45.6	43.3	81.4	80.6
Some college	25.5	24.3	17.8	18.4	3.2	4.0
Bachelor's degree	16.5	16.7	22.1	22.7	12.3	12.7
Advanced degree	9.1	10.1	14.5	15.6	3.1	2.7
Marital status						
Married	63.1	64.1	73.3	72.8	28.1	29.0
Not married	36.9	35.9	26.7	27.2	71.9	71.0
Race/ethnicity						
White non-Hispanic	69.6	70.6	70.8	69.9	71.2	72.2
Black non-Hispanic	13.8	11.6	12.2	11.0	11.6	10.2
Other	16.7	17.8	16.9	19.1	17.1	17.6
2001-2007 deployment^c^						
No	57.6	61.4	56.3	56.1	42.3	38.4
Yes	42.5	38.6	43.6	43.9	57.7	61.6
Military rank						
Enlisted	77.0	75.7	70.8	69.4	88.4	89.4
Officer	23.0	24.3	29.2	30.6	11.6	10.6
Service component						
Reserve/Guard	43.0	36.8	53.4	51.3	40.0	36.7
Active duty	57.0	63.2	46.6	48.7	60.0	63.3
Branch of service						
Air Force	29.0	25.7	30.3	24.7	26.6	17.9
Army	47.4	48.1	47.7	52.1	48.2	55.0
Navy/Coast Guard	18.5	20.6	18.1	18.8	16.9	16.8
Marine Corps	5.1	5.6	4.0	4.3	8.3	10.3
Occupational category						
Others	69.9	69.0	69.4	68.3	72.5	72.0
Combat specialists	20.0	21.2	19.2	20.3	15.7	19.1
Heath care specialists	10.4	9.8	11.4	11.4	11.8	8.9
General health^d^						
Very good/excellent	59.0	48.9	55.3	45.6	54.3	41.4
Good	30.3	35.1	34.5	37.4	33.1	37.8
Fair/poor	7.7	13.3	8.9	15.9	8.9	17.7
Missing	3.0	2.7	1.3	1.0	3.6	3.1

^a ^Includes participants who had a meaningful response to the open-ended question, "Do you have any concerns that are not covered in this survey that you would like to share?"

^b ^Percentages were rounded and may not sum to 100.

^c ^Any deployment in support of the wars in Iraq and Afghanistan September 2001-October 2007.

^d ^Self-reported general health from the question, "In general, would you say your health is excellent, very good, good, fair, or poor?"

The adjusted odds of response to the open-ended question for each of the respective response groups are displayed in Table 2. Increased adjusted odds of response to the open-ended question were found in personnel with service in the Army, Navy/Coast Guard, and the Marine Corps in comparison with Air Force members. Cohort members who were older, serving on active duty and in combat specialties were significantly more likely to respond to the open-ended question across all panels. Black non-Hispanic participants were significantly less likely to respond than white non-Hispanic participants. Among all panels, those who indicated fair or poor health were nearly three times more likely to respond when compared with those reporting very good or excellent health. Panel 1 women were more likely than men to provide a meaningful open-ended response, while no sex difference was observed among Panel 2 participants. Panel 1 baseline participants with deployment experience between 2001 and 2007 in support of the operations in Iraq and Afghanistan were less likely to respond to the open-ended question. However, Panel 1 follow-up and Panel 2 baseline participants with deployment experience in support of the operations in Iraq and Afghanistan were more likely to respond to the open-ended question.

**Table 2:** Adjusted Odds of Response to the Open-Ended Question by Characteristics of Millennium Cohort Study Participants

	Adjusted Odds of Response to Open-Ended Question^a^					
	Panel 1 Baseline n = 74,664		Panel 1 Follow-up *n *= 54,250		Panel 2 Baseline *n *= 29,902	
Characteristic	AOR	95% CI	AOR	95% CI	AOR	95% CI
Sex						
Male	ref		ref		ref	
Female	1.07*	1.02, 1.12	1.09*	1.03, 1.16	1.00	0.92, 1.07
Birth year						
Before 1960	1.00		1.00		1.00	
1960-1969	0.83*	0.79, 0.87	0.81*	0.76, 0.86	0.78	0.53, 1.15
1970-1979	0.65*	0.61, 0.96	0.71*	0.67, 0.76	0.64*	0.44, 0.93
1980 or later	0.52*	0.47, 0.58	0.57*	0.50, 0.65	0.49*	0.34, 0.71
Education						
High school or less	ref		ref		ref	
Some college	1.03	0.98, 1.09	1.09*	1.02, 1.16	1.33*	1.11, 1.59
Bachelor's degree	1.07	0.99, 1.15	1.13*	1.05, 1.22	1.17*	1.00, 1.37
Advanced degree	1.07	0.97, 1.18	1.17*	1.06, 1.29	1.15	0.88, 1.50
Marital status						
Married	ref		ref		ref	
Not married	1.09*	1.04, 1.14	1.06*	1.01, 1.12	1.06	0.98, 1.14
Race/ethnicity						
White non-Hispanic	ref		ref		ref	
Black non-Hispanic	0.71*	0.67, 0.75	0.82*	0.76, 0.88	0.80*	0.72, 0.90
Other	0.95*	0.90, 1.00	1.07*	1.00, 1.14	0.99	0.90, 1.08
2001-2007 deployment^b^						
No	ref		ref		ref	
Yes	0.88*	0.84, 0.91	1.13*	1.08, 1.19	1.10*	1.02, 1.18
Military rank						
Enlisted	ref		ref		ref	
Officer	1.07	0.99, 1.15	1.05	0.97, 1.14	1.06	0.88, 1.27
Service component						
Reserve/Guard	ref		ref		ref	
Active duty	1.50*	1.44, 1.57	1.14*	1.09, 1.20	1.32*	1.22, 1.43
Branch of service						
Air Force	ref		ref		ref	
Army	1.30*	1.24, 1.38	1.43*	1.35, 1.52	1.72*	1.57, 1.88
Navy/Coast Guard	1.26*	1.18, 1.34	1.35*	1.26, 1.45	1.39*	1.24, 1.55
Marine Corps	1.42*	1.30, 1.56	1.56*	1.38, 1.76	1.82*	1.59, 2.08
Occupational category						
Others	ref		ref		ref	
Health care specialists	0.90*	0.84, 0.96	1.00	0.93, 1.08	0.76*	0.67, 0.86
Combat specialists	1.07*	1.02, 1.13	1.08*	1.02, 1.15	1.18*	1.07, 1.29
General health^c^						
Very good/excellent	ref		ref		ref	
Good	1.55*	1.49, 1.61	1.47*	1.39, 1.54	1.60*	1.48, 1.72
Fair/poor	2.66*	2.50, 2.84	2.79*	2.59, 3.00	3.08*	2.79, 3.41

*Indicates statistical significance at the α = 0.05 level, with a 95% confidence interval that excluded 1.00.

^a ^Includes participants who had a meaningful response to the open-ended question, "Do you have any concerns that are not covered in this survey that you would like to share? A separated logistic regression model was run for panel 1 baseline, panel 1 follow-up, and panel 2 baseline populations.

^b^Any deployment in support of the wars in Iraq and Afghanistan September 2001-October 2007.

^c ^Self-reported general health from the question, "In general, would you say your health is excellent, very good, good, fair, or poor?"

Table 3 shows some example responses, as well as their associated clusters. Each row represents one cluster, with an example participant response displayed. Although the illness/injury cluster includes both chronic and acute concerns, blood pressure medication was the most commonly expressed issue. Exposure concerns were mostly either workplace hazards (e.g. toxic chemicals) or deployment concerns (e.g., being around strange chemicals during deployment). The responses classified in the exercise cluster mainly focused on fitness, although some responses overlapped between exercise and injury. Mental health included a wide range of responses, from childhood abuse to concerns about postdeployment readjustment. Although not readily apparent using human analysis, anxiety was identified as a separate cluster from mental health using LSA. Vaccination concerns were frequently expressed, even though the structured questionnaire contained a few vaccine questions.

**Table 3:** Example Responses From Millennium Cohort Study Participants Within the Top Seven Concerns Expressed in the Open-Ended Question

Area of Concern^a^	Example Response
Illness/injury^b^	I recently had my blood pressure medication dose increased to control hypertension
Illness/injury	I was involved in a motor vehicle collision...It has caused delays in my return to reserve duty/flight duty. I suffered a head injury/laceration and orthopedic injury/laceration to left knee.
Exposure	Exposed to hepatitis, asbestos, and enriched uranium in Uzbekistan and Afghanistan.
	Exposure to welding fumes.
Exercise	Lower back, knee, and ankle pain due to extended periods of massive weight-bearing duties and exercise.
Mental health	Mental and emotional problems due to sexual child abuse.
Anxiety/disorientation	Extreme stress and anxiety due to superiors' incompetence.
Vaccination	Allergic reactions to anthrax vaccine.

^a ^A single participant response could be categorized into multiple areas of concern.

^b^The cluster labeled "illness/injury" describes responses across a broad number of concerns. Several examples are provided in Table 4 to better illustrate these topic areas within the cluster.

The most frequently expressed areas of concern are shown in Table 4. Responders to the open-ended question most frequently expressed a concern with an illness or injury (28.0 percent). Terms present in the response that represented illness or injury concerns included words such as "suffered," "recovered," and "developed." Some of the other more frequently expressed areas of concern were exposure, discussed in 13.6 percent of open-ended responses and indicated by words such as "chemicals," "radiation," and "asbestos"; and exercise, discussed in 11.0 percent of open-ended responses, represented by terms such as "walking," "biking," and "vigorous". Other common concerns were back pain (8.8 percent), deployment (7.6 percent), arm symptoms (7.4 percent), mental health (7.2 percent), weight (6.3 percent), vaccination (4.5 percent), anxiety/disorientation (3.5 percent), and surgery (2.1 percent). Panel 1 open-ended responders more frequently expressed concerns about deployment at follow-up (8.3 percent) compared with baseline (7.1 percent). Compared with the total study population, a greater proportion of Panel 1 follow-up and Panel 2 baseline responders, who both filled out their respective survey from 2004-2006, indicated concerns about deployment and mental health.

**Table 4:** Most Frequently Expressed Areas of Concern Among Millennium Cohort Study Participants Responding to the Open-Ended Text Question

Area of Concern^a^	Total *n *= 10,214		P1 Baseline Responses *n *= 5,626		P1 Follow-up Responses *n *= 3,297		P2 Baseline Responses *n *= 1,291		Related Terms^b^
	***n***	**%**	***n***	**%**	***n***	**%**	***n***	**%**	

Illness/injury	2,859	28.0	1,433	25.5	1,033	31.3	393	30.4	suffered, recovered, developed
Exposure	1,385	13.6	887	15.8	328	10.0	170	13.2	chemicals, radiation, asbestos
Exercise	1,125	11.0	613	10.9	391	11.9	121	9.4	walking, biking, vigorous
Back pain	903	8.8	482	8.6	313	9.5	108	8.4	discs, herniation, lumbar
Deployment regions/concerns	780	7.6	399	7.1	275	8.3	106	8.2	Bosnia, barracks, DU
Arm	754	7.4	465	8.3	225	6.8	64	5.0	elbow, pronate, grip
Mental health	735	7.2	385	6.8	240	7.3	110	8.5	emotional, interpersonal, anxiety
Weight concerns	647	6.3	354	6.3	206	6.3	87	6.7	lose, dieting, obesity
Vaccination	459	4.5	299	5.3	114	3.5	46	3.6	VAERS, influenza, boosters
Anxiety/disorientation	355	3.5	185	3.3	117	3.6	53	4.1	shortness, sweating, tiredness
Surgery	212	2.1	124	2.2	55	1.7	33	2.6	removed, tape, wrapped

^a^Participants were able to provide a response in more than one area at multiple time points

^b^Example terms included in the same cluster that is described by the Area of Concern

## Discussion

As computing capabilities grow, researchers are increasingly given opportunities to use complex and computationally intensive analytic techniques to answer scientific questions. Confronted with practical challenges of analyzing open-text responses, LSA offers a comprehensive method for efficient and standardized analysis of these data. In this exploratory analysis, we found subgroups of the population that were more likely to use the open-text response option. Of greatest interest are those who reported poor general health and their propensity to use the open-text field. Since these individuals may be of high concern in health research, this text field yields additional valuable insight not otherwise assessed.

Limited research exists on the characteristics of individuals who choose to provide additional information as part of an optional open-ended text field on a survey. The strongest association observed in this study was that participants with poorer self-reported general health were significantly more likely to respond within the open-ended text field, and the likelihood of response increased as self-reported health status decreased. Interestingly, in the entire Millennium Cohort, it has been shown that there is not a significant association between health status and likelihood of enrollment [10]. However, it is important to note that all of the individuals in this current study were already participants in the Millennium Cohort Study; therefore, even though they may not have enrolled based on their health status, perhaps health status motivated them to provide additional information in the open-ended field. Those with poor self-perceived general health may be more likely to report symptoms [11], or perhaps they have a desire to explain their poor health in greater detail than do healthier individuals. Regardless of why individuals with poorer self-reported general health are more likely to respond to the open-ended question, this finding should be considered when conducting future analyses of response bias in the Millennium Cohort.

With nearly 1 in 5 respondents choosing to include information in the open text field, it is important to know their characteristics. Adjusted data interestingly suggest some weak patterns, albeit significant, in response to the open text field differentiated by sex, age, active-duty status, and combat occupations. Air Force personnel were least likely to include a meaningful response to the question, but were also most likely to respond and respond early to the initial invitation for enrollment [6,12]. Combat specialists and Marine Corps members were also more likely to respond to the open text question, which may be attributable to the ongoing combat operations in Iraq and Afghanistan. Other findings of education status indicate that response rates generally increase as education level increases; this does not hold true for the open ended response. This non effect could be attributed to the free form nature of the open-ended text field; reading comprehension of the participant may be less of an issue when compared with the structured instrument.

Another interesting finding is that illness/injury was by far the most frequently expressed area of concern. This may suggest that physical or emotional ailments cause concern for people; either about how or why illness or injury occurred, or how these ailments may affect their short- or long-term quality of life. It is also worth noting that a higher proportion of individuals reported concerns regarding either illness/injury or deployment on the 2004-2006 assessment compared with the 2001-2003 assessment. This may be a reflection of the increased deployments to Iraq and Afghanistan as the conflicts continued to heighten over this time period. With only one follow-up data point available for the present study, it was difficult to fully understand this relationship; however, it will be interesting to examine whether these concerns persist at the same or increased levels in the 2007-2008 and future assessments.

The Millennium Cohort Study team re-examines the structured survey instrument between survey cycles, frequently adding questions that were not originally included in the previous instrument. Based in part on the open-ended text analysis described in this paper, several changes have been made: in 2004, physical activity questions were added to the survey; in 2007 questions were added that focused on physical injury and deployment-specific exposures; in 2010, the physical injury section was supplemented, and questions on sleep length and quality were included. There was a very small proportion of responses related to very specific chemical exposures or other topics that were outside the scope of the survey, or very specific to a few individuals. The open ended question allows a channel for participants to raise awareness of newly identified, cutting edge topics that can help inform survey designers.

There are some limitations to these analyses that should be mentioned. The study population consisted of a sample of responders to the Millennium Cohort questionnaire and may not be representative of the military population. However, investigations of potential biases in the Millennium Cohort have found a well-representative military cohort who report reliable data and who are not influenced to participate by poor health prior to enrollment [6,10,13-20]. Latent Semantic Analysis is a technique to transform qualitative data into quantitative information, but it has limitations, including situations where meaning is determined contextually. Additionally, it is possible that non obvious underlying relationships existed within the top-20 automatically generated clusters, which could reveal more concerns that we were unable to detect. While these clusters were not included in the attached tables, they were included in the demographic analysis. The greatest limitation to using LSA on open-ended text responses, however, is the vagueness in grouping certain responses together. LSA approximates semantic meaning (related concerns) by using mathematical transformations as a proxy; not all mathematically related responses were obviously similar. This made it more difficult to cleanly distinguish between different clusters when performing the final analysis.

Despite these limitations, there are important strengths of this analysis. To our knowledge, this study is one of the first to apply LSA-based analyses to open-ended epidemiologic survey responses from a large US military population. This is also one of the first studies to examine the open-ended text responses from US military personnel, including reserve/National Guard, and members who have left military service. Previous analyses on military populations used human assisted computer analysis, but generally had less sophisticated methodologies [21]. Once the initial semantic space is created, LSA is fully automatic, permitting rapid analysis of large sets of responses. Because knowledge of word meaning is not derived from thesauri, ontologies, or hand-coding of relationships among words or among responses, bias from human coders and interpretation error is minimized. LSA can evaluate a word whose meaning is determined contextually (e.g., "we moved back," is differentiated from "hurt my back"). Furthermore, it can determine similarity among responses without accounting for word order or even if passages share no words in common [22]. We also examined the reliability of LSA versus human expert review of a random sample of 50 open-ended responses using the Kappa coefficient [23], and found agreement between LSA and human review to be substantial to almost perfect for four out of five categories examined, bolstering confidence in the LSA technology.

## Conclusion

Future directions of this work may include application of analyses to better define concerns within the Cohort. Comparisons between the structured response and open-ended sections could be used to evaluate the comprehension of the structured instrument. Open-ended text can reveal additional issues of prominent importance to participants. Investigators are continually challenged with addressing symptom-based illness that may not be well-defined under previous disease paradigms, and open-ended responses among large populations are critical to understanding such complex syndromes [24]. In addition, as society increasingly prefers brief, text-based communication for many health issues, analyses of written messages among populations may reveal important public health trends [25]. Computerized text-parsing tools such as LSA allow an objective review of text responses that would be otherwise impossible to standardize. LSA may be used to define health concerns with related context, and identify whether they represent large-scale concerns of a few individuals or common concerns of a great many individuals. Results will continue to help drive directions of future research and survey content. Review of open-ended text with text-mining tools such as LSA is critical to allow participant voices to truly be heard, from within the bounds of large-scale epidemiologic survey studies.

## Competing interests

The authors declare that they have no competing interests.

## Authors' contributions

All authors worked together on the conception, design, and methodology of the study. PF and MD performed the Latent Semantic Analysis and computerized text processing. IJ and CL performed the statistical analyses. TL, IJ, CL, BS, PA, MR, and TS participated in the interpretation of the analyses. TL, IJ, CL, BS, PF, MD, MR, and TS all participated in the authorship of the manuscript. All authors read and approved the final manuscript.

## Pre-publication history

The pre-publication history for this paper can be accessed here:

http://www.biomedcentral.com/1471-2288/11/136/prepub
